# Desmin forms toxic, seeding-competent amyloid aggregates that persist in muscle fibers

**DOI:** 10.1073/pnas.1908263116

**Published:** 2019-08-01

**Authors:** Niraja Kedia, Khalid Arhzaouy, Sara K. Pittman, Yuanzi Sun, Mark Batchelor, Conrad C. Weihl, Jan Bieschke

**Affiliations:** ^a^Department of Biomedical Engineering, Washington University in St. Louis, St. Louis, MO 63130;; ^b^Department of Neurology, Washington University School of Medicine, St. Louis, MO 63110;; ^c^Department of Energy, Environmental and Chemical Engineering, Washington University in St. Louis, St. Louis, MO 63130;; ^d^University College London Institute of Prion Diseases/Medical Research Council Prion Unit, University College London, London W1W 7FF, United Kingdom

**Keywords:** desmin, amyloid, myofibrillar myopathy

## Abstract

Desmin-associated myofibrillar myopathy (MFM) has pathologic similarities to neurodegeneration-associated protein aggregate diseases. Desmin is an abundant muscle-specific intermediate filament, and disease mutations lead to its aggregation in cells, animals, and patients. We reasoned that similar to neurodegeneration-associated proteins, desmin itself may form amyloid. Desmin peptides corresponding to putative amyloidogenic regions formed seeding-competent amyloid fibrils. Amyloid formation was increased when disease-associated mutations were made within the peptide, and this conversion was inhibited by the anti-amyloid compound epigallocatechin-gallate. Moreover, a purified desmin fragment (aa 117 to 348) containing both amyloidogenic regions formed amyloid fibrils under physiologic conditions. Desmin fragment-derived amyloid coaggregated with full-length desmin and was able to template its conversion into fibrils in vitro. Desmin amyloids were cytotoxic to myotubes and disrupted their myofibril organization compared with desmin monomer or other nondesmin amyloids. Finally, desmin fragment amyloid persisted when introduced into mouse skeletal muscle. These data suggest that desmin forms seeding-competent amyloid that is toxic to myofibers. Moreover, small molecules known to interfere with amyloid formation and propagation may have therapeutic potential in MFM.

## Significance

Protein aggregation and the deposition of amyloid is a common feature in neurodegeneration, but can also be seen in degenerative muscle diseases known as myofibrillar myopathies (MFMs). Hallmark pathology in MFM patient muscle is myofibrillar disarray, aggregation of the muscle-specific intermediate filament, desmin, and amyloid. In some cases, a missense mutation in desmin leads to its destabilization and aggregation. The present study demonstrates that similar to neurodegenerative proteins, desmin can form amyloid and template the amyloidogenic conversion of unaggregated desmin protein. This desmin-derived amyloid is toxic to myocytes and persists when introduced into skeletal muscle, in contrast to unaggregated desmin. These data demonstrate that desmin itself can form amyloid and expand the mechanism of proteinopathies to skeletal muscle.

Myofibrillar myopathies (MFMs) are a family of genetically defined degenerative myopathies that are due to mutations in 2 categories of proteins: Z-disk elements or protein chaperones (1). Specifically, disease mutations in sarcomeric structural proteins that reside at the Z-disk such as desmin, myotilin, filamin C, and Z-band alternatively spliced PDZ-motif lead to their accumulation and aggregation within myofibers. In addition, dominantly inherited mutations in protein chaperones necessary for the proper folding and assembly of these structural proteins such as αB-crystallin, BAG3, and DNAJB6 lead to myofibrillar disorganization and accumulation of sarcomeric proteins such as desmin. MFM patient muscle is unified by characteristic features that include muscle degeneration, protein inclusions, congophilic amyloid, and the aggregation of myofibrillar proteins such as desmin and myotilin (1, 2).

Desmin is a cardiac- and skeletal muscle-specific type III intermediate filament (IF) that is necessary for both myofiber and myofibril organization (3). Desmin inclusions are present in pathologic myofibers from patients with MFM (2), and are a principal component of protein inclusions in other protein aggregate myopathies not due to desmin mutations including acquired myopathies, such as sporadic inclusion body myositis (4). Dominant or recessively inherited missense mutations or in-frame deletions in desmin lead to a range of phenotypic syndromes that can include distal myopathy, limb girdle weakness, congenital weakness, and cardiomyopathy, yet all are unified by desmin aggregation within the affected tissue (3).

Desmin is a 470-amino acid protein, and more than 70 disease-associated mutations have been reported that span the entire protein (3). The formation of desmin IFs occurs via sequentially ordered steps that include dimer and tetramer formation, unit-length filament formation, and filament elongation (5). Some disease mutations affect IF assembly in vitro and in vivo, resulting in cytosolic inclusions (5, 6). Similarly, disease mutations in the small heat shock protein αB crystallin affect its ability to facilitate desmin filament formation, resulting in desmin aggregation (7). Desmin aggregates may directly affect myofiber function by disrupting sarcomere architecture (6). Alternatively, the accumulation of aggregated desmin may affect protein homeostasis, resulting in chaperone deficiency, proteasome impairment, mitochondrial dysfunction, and up-regulation of autophagy (8–11).

Some studies have demonstrated that desmin aggregates colocalize to congophilic inclusions or to antibodies detecting preamyloid oligomers (12–14). These studies suggest that desmin may indeed be amyloidogenic, similar to other aggregate-prone proteins in neurodegeneration such as microtubule-associated protein tau in Alzheimer’s disease and α-synuclein in Parkinson’s disease. In normal central nervous system tissue, these proteins are soluble, but in the setting of disease, they aggregate and become insoluble (15). In vitro, tau and α-synuclein form amyloid fibers, which formation is enhanced when missense mutations associated with disease are introduced (15).

Self-assembly of amyloidogenic proteins generally follows a mechanism of nucleated polymerization (16, 17). In this mechanism, de novo amyloid formation requires one or more thermodynamically unfavorable steps during nucleation. Nucleation results in sigmoidal assembly kinetics that are characterized by an initial lag-phase. As a general feature of nucleated polymerization, preformed amyloid structures can template or seed the conversion of unaggregated and soluble monomeric protein into the amyloid fold. Amyloid structures can replicate if templated conversion is coupled with the generation of new seeds; for example, by fibril breakage (17, 18). Replication via seeded polymerization is thought to underlie the spread of aggregate pathology within cells and its transmission between cells and organisms in multiple neurodegenerative diseases, such as Creutzfeldt-Jakob, Parkinson’s, and Alzheimer’s disease.

While several studies have demonstrated that diseased skeletal muscle can accumulate aggregate-prone proteins known to form amyloid in vitro, such as β-amyloid (Aβ) and gelsolin (19), there is no evidence that a MFM-associated protein is amyloidogenic, or that it can replicate by a seeding mechanism. The following study explores the amyloidogenicity of the muscle-specific protein desmin. We suggest that desmin amyloids are the culprit of myofiber degeneration and that they may share other amyloid properties, such as seeding, and may be important mediators of MFM pathogenesis.

## Results

### Aggregation of Desmin Fragments into Seeding-Competent Amyloid Fibrils.

**Fig. 1.**
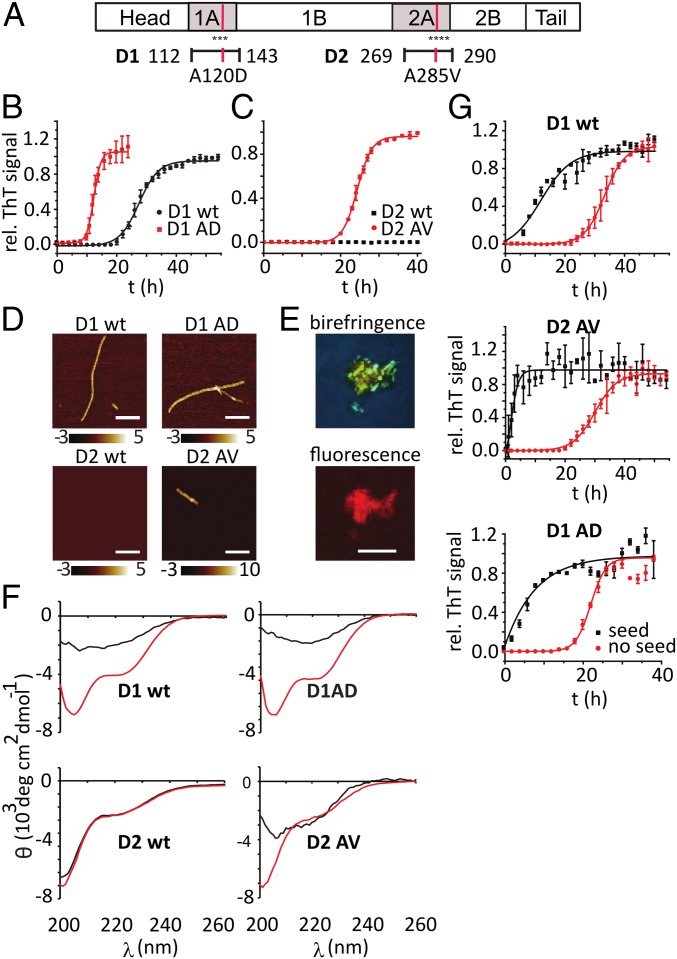
(*A*) Schematic diagram of full-length human desmin protein with amyloidogenic regions D1 and D2. (*B* and *C*) ThT aggregation kinetics of D1wt and D1 A120D peptides (*B*), and D2 wt and D2 A285V (*C*), respectively; mean normalized ThT fluorescence ± SD, *n* = 3. (*D*) AFM images of aggregated D1wt, D1AD, D2wt, and D2AV. D2wt did not form any fibrils. (Scale bar, 350 nm.) (*E*) CR birefringence and fluorescence of D1wt peptide aggregates. (Scale bar, 10 µm.) (*F*) CD spectra of D1wt, D1AD, D2wt, and D2AV peptides incubated for 0 h (red) and 3 d (black), as in *B*. (*G*) Aggregation kinetics of D1wt D1AD and D2AV (0.3 mg/mL), respectively, in the presence of preformed seeds (0.03 mg/mL). All assays at 37 °C in 20 mM glycine buffer, 100 mM NaCl at pH 2.8.

We analyzed the amino acid sequence of desmin in silico for its amyloid propensity, using the prediction algorithm Waltz (20). Waltz identified 2 highly amyloidogenic regions, from aa 118 to 124 and from aa 280 to 290 (Fig. 1 *A*, marked by asterisks). Both regions contained mutations that cause MFM; specifically, A120D in the first region and A285V in the second region (21, 22). The CamSol algorithm predicted that the A285V mutation increased hydrophobicity and reduced solubility of the region (23).

To test whether these regions of desmin could indeed form amyloid, we purified synthetic peptides that spanned either region (D1, aa112 to 143; D2, aa269 to 290) and that contained either the wt sequence (D1wt and D2wt) or the disease mutations A120D or A285V, respectively (D1AD and D2AV). These peptides were incubated in a wide range of pH conditions to probe their aggregate formation by thioflavin T (ThT) fluorescence (*SI Appendix*, Fig. S1*A*). We observed sigmoid kinetics that are typical for amyloid formation for the D1 peptide (Fig. 1*B*); the disease mutation A120D accelerated aggregation (Fig. 1*B*). Similarly, the D2AV peptide formed ThT-positive aggregates, while the D2wt peptide remained soluble (Fig. 1*C*). Atomic force microscopy (AFM) revealed that all aggregated peptides formed fibrils. D1wt fibrils had an average height of 4 ± 0.2 nm and a repetitive height profile that suggests a helical substructure with a repeat length of 32 ± 2 nm (Fig. 1*D* and *SI Appendix*, Fig. S1 *B*–*D*); D1AD and D2AV fibrils had similar dimensions to the D1 wt fibrils.

Birefringence of the amyloidophilic dye Congo red (CR) is a hallmark of amyloid structures. The aggregated peptides bound CR and displayed both CR fluorescence and apple green birefringence when observed under cross-polarized light (Fig. 1*E* and *SI Appendix*, Fig. S2).

We probed the secondary structure of the desmin peptides by circular dichroism (CD) to test whether they form β-sheet structures under our experimental conditions (Fig. 1*F*). The spectra of all 4 peptides had minima at 200 nm, typical for unstructured polypeptides. After 3 d incubation, D1wt, D1AD, and D2AV peptide spectra showed transitions to a β-sheet structure with minima at 218 to 220 nm (Fig. 1*F*), while the D2wt peptide remained unstructured.

To confirm that amyloid fibers can act as seeds that accelerate fibril formation, we generated fibril fragments by sonication. The aggregation of all 3 peptides was strongly accelerated by addition of these seeds (Fig. 1*G*). We used the analytical framework developed by Tuomas Knowles et al. to analyze whether seeding occurred either via elongation/fragmentation (i.e., a prion mechanism) or via secondary nucleation (24). We analyzed the concentration dependence of D1wt, D1AD, and D2AV peptide fibril formation in the AmyloFit algorithm, using models of nucleated polymerization that included only primary nucleation, primary and secondary nucleation, primary and secondary nucleation, and prion replication (*SI Appendix*, Fig. S3). The data were best described by a model in which prion-like replication dominated the aggregation kinetics; however, seeding by secondary nucleation may equally contribute to fibril formation. These results all indicate that fragments from the MFM-associated regions of desmin form seeding-competent, β-sheet rich amyloid fibrils.

### Disease Mutations Lower the Critical Concentration of Fibril Formation.

To quantify the role of disease-associated mutations A120D and A285V in amyloid formation, we analyzed their effect on the critical concentration in nucleated polymerization (16). The critical concentration of a polypeptide reflects its amyloidogenicity (24). We separated soluble from insoluble peptides by ultracentrifugation (20 min, 100,000 × *g*) and quantified the peptide in the total (T) and supernatant (S) fractions by SDS/PAGE and Coomassie staining (*SI Appendix*, Fig. S4*A*). The graphs in *SI Appendix*, Fig. S4 *B*–*D* represent the densitometric analysis of soluble vs. total peptides normalized to the respective highest peptide concentration. As predicted by the model of nucleated polymerization, the amounts of soluble peptide remained constant, regardless of the initial concentration. The relative critical concentrations of the 3 peptides mirrored their respective aggregation kinetics, with the fastest-aggregating peptide D1AD having the lowest critical concentration (0.16 ± 0.05 mg/mL, 50 µM), followed by D1 wt (0.3 ± 0.03 mg/mL, 77 µM) and D2AV (0.28 ± 0.06 mg/mL, 121 µM). These data suggest that the desmin peptide aggregation followed a nucleated polymerization mechanism, and that the disease-associated mutations A120D and A285V increased amyloid formation.

### Anti-Amyloid Compound EGCG Slows the Aggregation of Desmin Fragments.

**Fig. 2.**
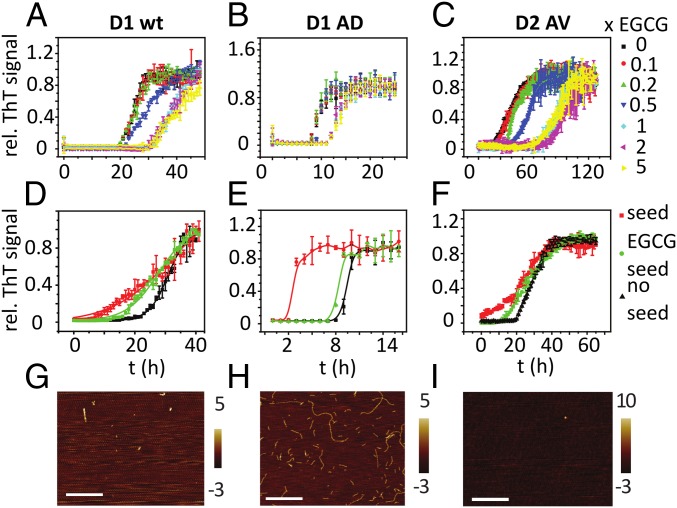
(*A*–*C*) Aggregation of D1wt, D1AD, and D2AV in the presence of different molar ratios of EGCG (*D*–*F*), as in Fig. 1. Peptides incubated in the presence of EGCG (5:1 molar ratio) were collected as seeds (D1 wt, 24 h; D1AD, 9 h; D2 AV, 60 h); mean normalized ThT fluorescence ± SD, *n* = 3. (*D*–*F*) Monomeric peptides were then aggregated without seeds (black), with fibrillar seeds (10% wt/wt, red) or with seeds formed in presence of EGCG (green). Seeds formed in the presence of EGCG only weakly accelerated aggregation kinetics. AFM image of peptide + EGCG seeds for D1wt (*G*), D1AD (*H*), and D2AV (*I*). (Scale bars, 600 nm.)

Numerous studies have demonstrated that the polyphenol epi-gallocatechin gallate (EGCG) is a potent inhibitor of seeding-competent amyloid fibrils. Desmin peptides D1wt, D1AD, and D2AV were incubated with EGCG at molar ratios of 0.1 to 5× (EGCG: peptide) to test its effect on desmin fibril formation. EGCG delayed formation of ThT binding fibrils in a concentration-dependent manner (Fig. 2 *A–C*). However, the compound did not prevent fibril formation, as we previously observed for Aβ and for α-synuclein (25). To test its effect on seeding, we collected seeds in the prenucleation phase of desmin peptides that were incubated with EGCG at equimolar concentration (D1wt, 29 h; D1AD, 11 h; D2AV, 65 h incubation). When added to fresh monomeric peptide, peptides incubated in the presence of EGCG displayed much decreased seeding activity compared with peptide samples that were incubated in the absence of EGCG (Fig. 2 *D* and *E*). From these data, we conclude that EGCG delayed but did not prevent seed formation at 5× molar concentration (Fig. 2 *G* and *H*).

With Aβ and α-synuclein, EGCG induces the formation of spherical, SDS-stable aggregates that prevent seeding of amyloid fibrils (25) However, atomic force microscopy gave no indication that EGCG induced the formation of spherical aggregates of desmin fragments. When we probed the formation of SDS-stable aggregates in the presence of EGCG by SDS/PAGE, we observed no indication of stable aggregates in the presence of SDS (*SI Appendix*, Fig. S5). These results indicate that, at our experimental conditions, the effect of EGCG on desmin more closely resembled the tau protein rather than Aβ and α-synuclein aggregation, as the compound delayed, but did not prevent, nucleation of desmin peptides at low pH.

### Desmin Fragment 117 to 348 Forms Seeding-Competent Amyloid Fibrils under Physiologic Conditions.

**Fig. 3.**
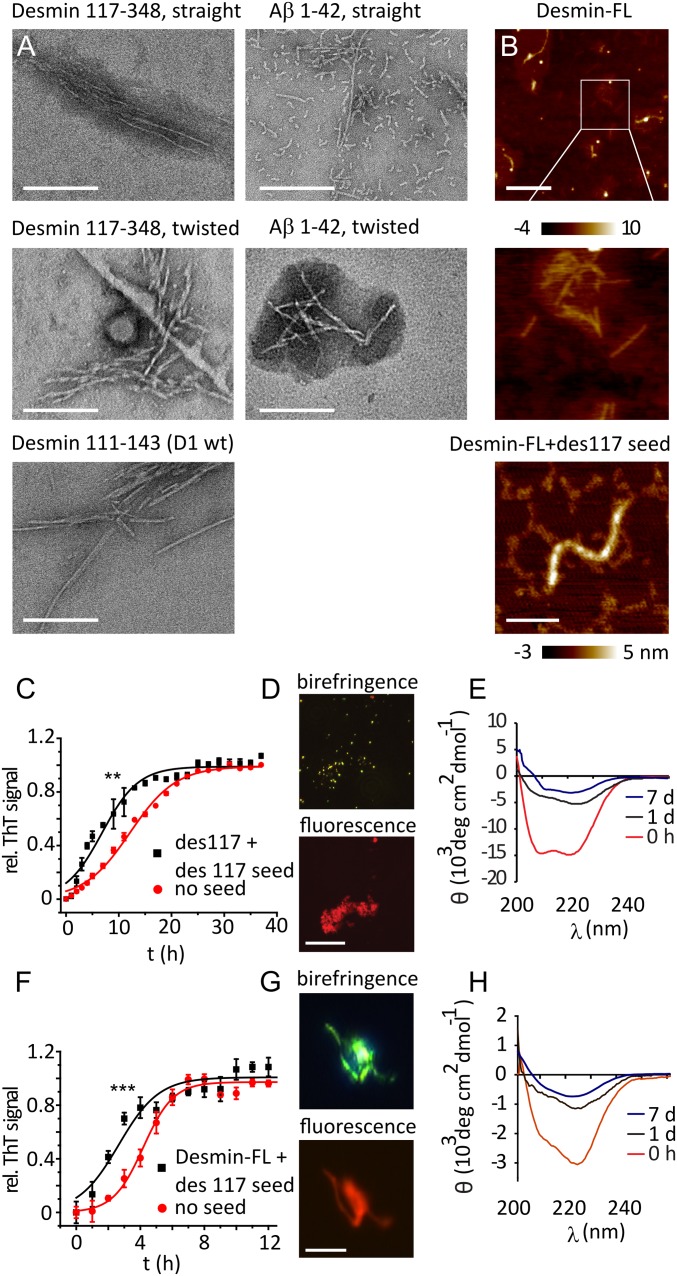
(*A*) Negative stain transmission electron microscopy images of Desmin 117, D1 wt, and Aβ 1 to 42 fibrils. (Scale bars, 200 nm.) Desmin formed narrow, straight fibrils and twisted fibrils with more than one filament. Both morphologies are also observed in Aβ 42. Fibril diameters were measured using ImageJ; mean ± SD, *n* = 20. (*B*) AFM images of full-length desmin fibrils in the absence and presence of 10% fibrillar des117 seeds. (*Middle*) Highlighted area containing straight fibrils of ∼6 nm height. (Scale bar, *Top*: 400 nm; *Bottom*: 100 nm.) (*C*) Aggregation of desmin 117 to 348 peptide (des117, 0.55 mg/mL) 10% fibrillar des117 seeds; mean normalized ThT fluorescence ± SD, *n* = 3. (*D*) Des117 aggregate CR fluorescence and birefringence. (Scale bar, 25 µm.) (*E*) CD spectra of des117 at 0 h and after 1 d of aggregation. (*F*) Aggregation kinetics of full-length desmin with 10% fibrillar des117 seeds. (*G*) Desmin aggregate CR fluorescence and birefringence. (Scale bar, 25 µm.) (*H*) CD spectra of full-length desmin after 0 h and 1 and 7 d incubation. All reactions (*A*–*H*) at 37 °C in 50 mM NaP buffer at pH 7.4, 100 mM NaCl. **P < 0.001; ***P < 0.0001.

Short desmin peptides of either the D1 or D2 region formed amyloid fibrils only under acidic conditions. However, longer protein fragments may be able to form amyloid fibrils under physiological conditions. We tested this hypothesis by incubating the desmin fragment 117 to 348 that spans both amyloidogenic regions (des117) in 50 mM Na-phosphate buffer (NaP) at pH 7.4, 100 mM NaCl. Strikingly, the des117 fragment formed ThT-positive amyloid (Fig. 3 *A–C*) that displayed CR fluorescence and birefringence (Fig. 3*D*).

Electron microscopy revealed that the polypeptide formed 2 types of fibrils with distinct morphologies: narrow straight fibrils (6.3 ± 0.5 nm diameter), similar to the short desmin peptide D1, and twisted filaments with 9.0 ± 0.8 nm diameter (Fig. 3*A*). Similar morphologies were observed in amyloid-β fibrils (Fig. 3*A*). Both types of fibrils had similar average lengths of 150 ± 90 nm and 120 ± 70 nm, respectively. CD spectra showed a transition from a mostly α-helical structure (26) to an increasingly β-sheet-like spectrum after 1 and 7 d incubation (Fig. 3*E*).

However, unlike the short desmin fragments, aggregation kinetics of des117 were not dominated by prion-like seeding in vitro at our experimental concentrations (*SI Appendix*, Fig. S6 *A* and *B*). Rather, des117 aggregation displayed flat concentration dependence, similar to other folded proteins, such as prion protein and light chain amyloid formation (27–29). This could reflect a strong influence of conformational transition on the kinetics or the presence of alternative aggregation pathways.

Notably, des117 fibrils seeded not only aggregation kinetics of des117 monomer but also those of full-length desmin (Desmin-FL) into ThT-positive structures (Fig. 3 *B* and *F*). These desmin fibrils likewise displayed CR birefringence (Fig. 3*G*) and had diameters of ∼6 nm (straight fibril) or ∼10 nm (twisted fibril), typical of amyloid fibrils (Fig. 3 *A* and *B*), and CD spectra indicative of β-sheet secondary structure (Fig. 3*H*). In conclusion, these data strongly suggest that des117, similar to the shorter peptide fragments, forms amyloid fibrils that can seed amyloid formation of full-length desmin protein.

### Desmin Amyloid Is Toxic and Persists in Skeletal Muscle.

**Fig. 4.**
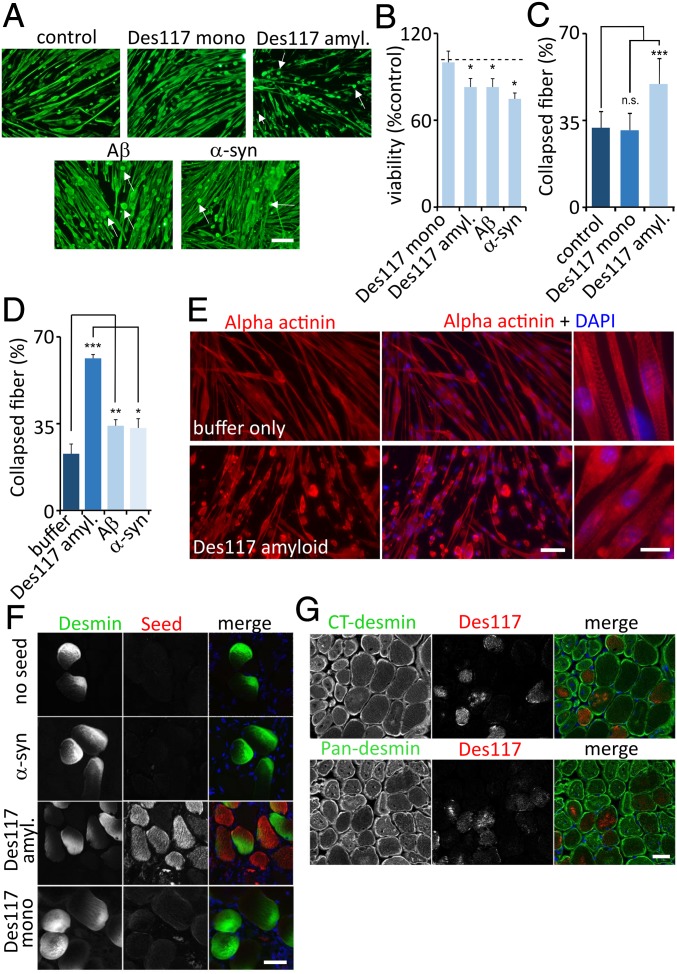
(*A*) Primary mouse myotubes treated with buffer, des117 monomer, des117 amyloid, or control amyloids (Aβ or α-synuclein) immunostained with an anti-desmin antibody. Arrows denote collapsed myofibers. (*B*) MTT viability assay of cells in *A* as a percentage of untreated control cells (*dashed line*); mean ± SD, *n* = 12. (*C*–*D*) Quantitation of the percentage of collapsed fiber in primary mouse myotubes treated with buffer, des117 monomer, des117 amyloid, Aβ 42 or α-synuclein (*n* = 10; >1,000 myotubes/condition). (*E*) Primary myotubes treated with buffer or des117 amyloid and stained with anti α-actinin antibody. Collapsed fibers have diffuse α-actinin staining, as opposed to elongated fibers with sarcomeric structure. (*F*) Confocal images of cryosections of tibialis anterior muscle electroporated with GFP-desmin and fluorescently labeled α-synuclein fibrils, des117 amyloid or des117 monomer. (*G*) Confocal images of cryosections of tibialis anterior muscle electroporated with fluorescently labeled des117 amyloid then stained with an antibody recognizing the C terminus or an epitope within the desmin fragment (pan-desmin). Blue is DAPI-stained nuclei. *P < 0.01; **P < 0.001; ***P < 0.0001; n.s., not significant. (Scale bars: *A*, *E*, *Left*, *F*, and *G*, 50 μm; *E*, *Right*, 10 μm.)

To see whether desmin amyloids were toxic to skeletal muscle, we differentiated primary mouse myoblasts into myotubes and transduced them with monomeric des117, des117 amyloid, or 2 control amyloids, α-synuclein and β-amyloid. Twenty-four hours after transduction, myotube viability and internal structure were analyzed. Myotubes treated with des117 amyloid or control amyloids had a reduction in viability compared with buffer-treated or des117 monomer control (Fig. 4 *A* and *B*). This is consistent with previous studies demonstrating that amyloidogenic peptides such as β-amyloid are detrimental to myotubes (30, 31). It was striking that, although viability was similarly decreased with different amyloids, des117 amyloid resulted in more rounded and collapsed myotubes that remained viable but had lost cytoskeletal integrity (Fig. 4 *A* and *C*). This collapse was specific for the amyloid structure of desmin, since monomeric des117 did not alter myotube morphology (Fig. 4*D*). Evaluation of the internal IF architecture with an antibody to α-actinin demonstrated striated sarcomeric structures in control-treated myotubes that were lost in the collapsed des117 amyloid-treated myotubes, which had a more diffuse and homogenous α-actinin distribution (Fig. 4*E*).

To explore the behavior of desmin amyloids on skeletal muscle in vivo, we electroporated mouse tibialis anterior skeletal muscle with similar amounts of fluorescently labeled monomeric des117, des117 amyloid, or, as a control amyloid, α-synuclein, along with an expression vector containing GFP-desmin. After 7 d of expression and recovery from the electroporation, we isolated the tibialis anterior and sectioned the muscle for confocal fluorescence microscopy (Fig. 4*F*). While desmin-GFP was expressed similarly in transduced fibers, only des117 amyloid remained within the fiber compared with α-synuclein or monomeric des117. Des117 amyloid did not colocalize with transduced GFP-desmin, suggesting that they did not incorporate into desmin IFs.

IF disruption or incorporation was also not apparent when we transiently transfected an expression vector containing an N-terminal GFP tagged desmin and transduced fluorescently labeled des117, aggregated α-synuclein, aggregated des117, or aggregated full-length desmin protein into SW13 cells (*SI Appendix*, Fig. S7*A*). SW13 cells are an adrenal carcinoma cell line that lacks type III IFs such as vimentin and desmin, allowing one to evaluate newly synthesized IF formation. In addition, desmin 117 amyloid did not coaggregate with GFP-labeled full-length desmin when using MFM disease-associated mutations GFP-desmin-D399Y or GFP-desmin-R350P that form sarcoplasmic aggregates when electroporated into mouse tibialis anterior in vivo (*SI Appendix*, Fig. S7*B*).

To further evaluate the localization of transduced des117 amyloid, we electroporated only fluorescently tagged des117 amyloid into muscle and performed fluorescent immunohistochemistry with 2 desmin antibodies. One antibody directed to the C terminus of desmin recognizes only endogenous desmin, since the exogenous desmin amyloid is truncated at amino acid 348. The second antibody recognizes both the des117 fragment and endogenous desmin. Similar to coexpression of exogenous desmin-GFP, des117 amyloid persisted in the sarcoplasm of the electroporated skeletal muscle fiber, but there was no apparent colocalization with endogenous FL-desmin within the fiber containing des117 amyloid seeds, confirming the previous results with GFP-desmin coexpression (Fig. 4*G*).

## Discussion

Pathogenic mutations in desmin lead to muscle diseases with prominent protein inclusions within affected myofibers (3). Mutations in several other muscle specific proteins also lead to myopathies with protein inclusions or MFMs (1). In desmin-associated MFM, a dominantly inherited missense mutation causes it to misassemble and aggregate. Inclusions in MFMs often stain with CR and can demonstrate birefringence, which is one of the hallmarks of amyloid structures (19). The current study demonstrates that desmin itself has amyloidogenic properties. Desmin amyloids can propagate the aggregation of naive, unaggregated, monomeric desmin. We suggest that amyloidogenic conversion of desmin may underlie the pathogenesis of MFMs.

Desmin is the primary IF in skeletal and cardiac muscle. Notably, all MFMs, regardless of genetic etiology, contain desmin inclusions, suggesting that desmin aggregates unify MFM pathogenesis (19). We reasoned that desmin may actually be an amyloidogenic protein with properties similar to other proteins in neurodegenerative disorders. Several algorithms have been developed to predict amyloidogenicity based on hydrophobicity, amino acid composition, and sequence (20, 23). Indeed, Waltz identified 2 amyloidogenic regions that contained amino acids mutated in MFM. Peptides corresponding to these regions made amyloid in vitro that was 8 to 10 nm in height, bound ThT, displayed CR birefringence, had predominant β-sheet structures, and were competent to accelerate fibril formation through seeding. Moreover, a larger desmin fragment 117 to 348 that spanned both regions aggregated into fibrils with similar properties under physiological conditions. These fibrils coaggregated with full-length desmin protein and could seed the formation of fibrils with amyloid-like properties in vitro. CR fluorescent-positive amyloid structures are a feature found in some muscle biopsies from patients with MFM. In some cases, congophilic inclusions seem to colocalize with desmin aggregates on serial sectioned muscle biopsies (14). However, whether desmin is the congophilic species is not known. Our results suggest that desmin and its fragments can form congophilic aggregates that persist in muscle fibers. The mechanism by which desmin seeds persist, while unaggregated desmin does not, is unclear. It is conceivable that once aggregated, desmin is not cleared by normal proteolytic pathways such as the proteasome or autophagy, or that protein chaperones necessary for this process fail to recognize desmin amyloids leading to their selective persistence (3).

Several studies have expressed an amyloidogenic protein in skeletal or cardiac muscle to model a protein aggregate myopathy. These include expression of an expanded poly-glutamine containing protein, amyloid precursor protein, and gelsolin (32–34). Interestingly, all these models develop aged onset muscle dysfunction with fiber degeneration, amyloid inclusions, and in some cases, secondary aggregation of other proteins suggesting that amyloidogenic proteins can lead to disease (33, 34). The accumulation of proteins known to aggregate and form amyloid in neurodegenerative disorders are also present in tissue from patients with MFM and include TDP-43 and β-amyloid (19, 35). However, mutations in these proteins have not been found in patients with MFM or other protein aggregate myopathies, suggesting that their accumulation is secondary (36). Proteomic studies have consistently identified desmin as one of the most abundant proteins found in protein aggregates in MFM and other aggregate myopathies not associated with desmin mutations (4, 37, 38). In addition, posttranslational modification of desmin into phosphorylated fragments has been suggested to enhance its amyloidogenicity in vivo (12).

The mechanism of nucleated polymerization allows for multiple competing pathways of nucleation and replication (17, 24). Our data suggest that the self-assembly of unfolded desmin fragments spanning the amyloidogenic regions of the protein is dominated by seed elongation and fragmentation, which is typical for prion replication. While accelerated by seeding, the amyloid formation of the natively folded desmin 117 to 348 does not show concentration dependence. This suggests its aggregation rate may be limited by a conformational change, as has been observed for other folded amyloidogenic proteins, such as antibody light chain and the prion protein (27–29). Star-shaped fibril clusters formed by full-length desmin, similar to those observed in huntingtin aggregation, suggest a role of nucleated branching in fibril formation.

Amyloid structures are believed to persist and replicate in skeletal muscle. Most notably, the form of the prion protein (PrP) found in infectious prions, PrP^Sc^, likely incubates in the skeletal muscle of cattle, where it can then transmit to uninfected animals resulting in bovine spongiform encephalopathy (39). It has also been shown that α-synuclein aggregates when injected into skeletal muscle induce central nervous system α-synuclein pathology and motor impairment (40). Interestingly, there was no α-synuclein pathology in the injected skeletal muscle (40). This is similar to our data demonstrating that only desmin amyloid seeds persisted in skeletal muscle, whereas α-synuclein seeds were labile. It is conceivable that there is tissue specific tropism for muscle-derived versus central nervous system-derived amyloid species. Our data, along with others, support that skeletal muscle is a suitable reservoir for the persistence and replication of amyloid structures by prion or prion-like mechanisms.

One feature of prion diseases is the pathogenic spread of aggregate pathology from cell to cell via templated aggregate conversion. Whether this process occurs in skeletal muscle is not known. Notably, this mechanism is distinct from the expression of a destabilized desmin variant that aggregates upon intermediate filament assembly. Our studies demonstrated templated conversion of full-length desmin by desmin amyloid, but this was not recapitulated in cells or skeletal muscle. Indeed, desmin amyloid did not incorporate into native desmin filaments and desmin aggregates, nor did they disrupt IF formation under the conditions tested. Exogenous desmin amyloid, similar to other amyloids, was toxic to myotubes in culture, suggesting that amyloidogenic species in themselves can lead to myofiber dysfunction. Unique to treatment with exogenous desmin amyloid, myotubes collapsed and lost sarcomere integrity. In addition, desmin amyloid persisted in mouse skeletal muscle compared with desmin monomers or α-synuclein aggregates. This discrepancy between in vitro and in vivo studies is intriguing and may relate to the unconstrained biophysical properties of proteins in solution versus in cells that have a complete proteostatic network of chaperones and clearance mechanisms. Alternatively, the substrate of desmin amyloid propagation in vivo may be a proteolytic fragment of desmin, which would not have been detected by GFP or antibody staining. A similar mechanism is observed in the seeded propagation of the huntingtin protein, light chain amyloid, and PrP^Sc^, all of which preferentially or exclusively incorporate proteolytically truncated forms of the protein (18, 41).

The spread of aggregate pathology could conceivably propagate within the myofiber corrupting the Z-disk of adjacent or nearby sarcomeres. Alternatively, a toxic aggregate may escape a diseased myofiber and be actively taken by an adjacent fiber. This mechanism may explain the large regions of aggregate pathology and myofiber atrophy in MFMs (19).

The aggregation of proteins into amyloid structures and their subsequent spread via prion replication could have profound implications for therapeutic strategies in myodegenerative diseases. Therapeutic intervention that inhibits amyloid formation and inactivates seeds could halt replication. While no such clinical therapy exists today in neurodegenerative diseases, model compounds, such as EGCG, have been highly effective in inactivating amyloid seeds and inhibiting replication in vitro by sequestering them into seeding-inactive aggregates (25, 28). However, its mechanism depends on the relative stabilities of aggregation intermediates, fibrils, and EGCG-induced aggregates (25, 28). Future studies in mouse models of desmin aggregation and propagation will have to determine whether EGCG is a promising model compound for desminopathy in vivo.

## Experimental Methods

### ThT Aggregation Assay.

Aggregation of synthetic desmin peptides (D1 wt, D1 AD, D2 wt, D2 AV, Watson Bio; 0.33 mg/mL) was monitored by ThT fluorescence in 20 mM glycine buffer with 100 mM NaCl in a fluorescent plate reader, as described previously (25, 28). For aggregation of full-length desmin and des117 aggregation (CloudClone), 50 mM NaP buffer at pH 7.4, 100 mM NaCl was used.

### Primary Myotube Assays.

Primary mouse myoblasts were differentiated into myotubes and then transduced with 0.05 µg/µL unaggregated des117, des117 amyloid, α-synuclein fibrils or β-amyloid fibers in fusion medium using Lipofectamine 2000 for 24 h and assessed for toxicity and morphology, as described in the *SI Appendix*, which also contains additional *SI Appendix*, *Experimental Methods*.

## Supplementary Material

Supplementary File
