# Supplementary material for: Desmin forms toxic, seeding-competent amyloid aggregates that persist in muscle fibers
Source: Proc Natl Acad Sci U S A. 2019 Aug 1;116(34):16835–40. doi: 10.1073/pnas.1908263116 (PMC6708308; doi:10.1073/pnas.1908263116)
Supplement: Supplementary File [file pnas.1908263116.sapp.pdf]

PNAS
www.pnas.org

Supplementary Information for

Desmin forms toxic, seeding competent amyloid aggregates that persist in muscle fibers

Niraja Kedia[1], Khalid Arhzhaouy[2], Sara K Pittman[2], Yuanzi Sun[3,4], Mark Batchelor[4], Conrad C Weihl[2]*, Jan Bieschke[1,4]*

1 Department of Biomedical Engineering, Washington University in St. Louis, St. Louis, MO

2 Department of Neurology, Washington University School of Medicine, St. Louis, MO

3 Department of Energy, Environmental and Chemical Engineering, Washington University in St. Louis, St. Louis, MO

4 UCL Institute of Prion Diseases / MRC Prion Unit, University College London, UK

Jan Bieschke, Conrad Weihl
Email: j.bieschke@ucl.ac.uk; weihlc@wustl.edu

This PDF file includes:

Supplementary text
Figs. S1 to S7
References for SI reference citations

www.pnas.org/cgi/doi/10.1073/pnas.1908263116

Supplementary Information Text

Experimental Section

Analysis of desmin sequence by WALTZ and CamSol: For Waltz, the full amino acid sequence for human desmin protein (Uniprot P17661) with a threshold of best overall performance and pH 7.0 was used. For CamSol intrinsic solubility was performed on QYETIAAKNIS (DES WT 280-290) or QYETIVAKNIS (DES MT 280-290) at pH 7.0.

Cell culture: SW13 cells were purchased from Sigma-Aldrich (87031801). Cells were cultured in Dulbecco's Modified Eagle Medium (DMEM) (Gibco; 11965-084), 10% FBS (Atlanta Biologicals; S10350), 50 IU penicillin and 50 µg/mL streptomycin (Invitrogen; 15 140). Cells plated in a 12 well dish transduced with sonicated seeds at a working concentration of 0.04 µg/µL in Optimem media and 1.5 µL Lipofectamine 2000 per final volume of 500 µL per well. 30 minutes later, cells were additionally transfected with 0.3µg mEmerald-desmin-N-18 plasmid (a gift from Michael Davidson (Addgene plasmid # 54060)) in Optimem and 0.75 µL Lipofectamine 2000 per final volume of 500 µL per well. 48 hours post transduction/transfection, media was aspirated off, washed with 1xPBS and fixed

with 100% ice-cold methanol for 10 minutes, washed again in 1xPBS and coverslips were mounted to slides using water-based mounting media and DAPI. Primary mouse myoblasts were grown to ~75% confluence in DMEM/F10 Medium supplemented with 10% FBS and 0.1 ng/ml Recombinant Human Fibroblast Growth Factor rhFGF (Promega) on µ-Slide 8 Well ibiTreat chamber slides (Cat.No: 80826) coated with collagen. To induce cell fusion, cells were grown in fusion medium (DMEM supplemented with 10% horse serum (Remel™ TermoFisher scientific) for 5 days. Differentiated myotubes were then transduced with 0.05 µg/µL unaggregated des117, des117 amyloid, α-synuclein fibrils or β-amyloid fibers in fusion medium using Lipofectamine 2000 for 24h. Myotubes were fixed with acetone/methanol (1:1) for Immunofluorescence or used for MMT viability assay (Invitrogen) following manufacturer instructions. Collapsed myotubes describe fibers that remain viable but have lost their cytoskeletal integrity. The rounded fibers still maintain MTT activity but are morphologically different from elongated myotubes. Collapsed myotubes percentage from non-treated or treated myotubes was calculated by counting the number of rounded myotubes from Desmin antibody stained pictures using ImageJ software. More than 1000 myotubes were counted per condition. Antibodies used were mouse anti-desmin (clone D33, Dako M0760) and rabbit anti-α-actinin (Abcam ab68617). Statistics was performed using excel software and significance using Student's paired t-test.

In vivo electroporation: Mice are anesthetized using 2.5% isoflurane. Hair covering the Tibialis Anterior (TA) was shaved, wiping exposed skin with ethanol, and the animals injected with endotoxin-free plasmid diluted to 30-50µg total plasmid (mEmerald-Desmin/WT, mEmerald-Desmin/R350P or mEmerald-Desmin/D399Y) and 100µM seeds in sterile PBS to a volume of 50µL. The plasmid and seeds are then injected into the belly of the TA using a 29 gauge x 1/2 needle. Immediately following injection, two-needle array electrodes are inserted into muscle longitudinally, relative to muscle fibers. In vivo electroporation parameters were the following: voltage, 100 V; pulse length, 50 ms; number of pulses, six; pulse interval, 200 ms; desired field strength, 200 V/cm, given by a BTX ECM830 Electro Square Porator. Needles are removed and lower leg is wiped again with ethanol. Procedure is repeated on contralateral TA. Mice are then allowed to recover and returned to home cage for post-procedure monitoring. Animals remain in home cage 7 days prior to muscle isolation. Animals are then euthanized with isoflurane and TA is excised and frozen for sectioning and analysis. All techniques were performed according to ASC/IACUC approved protocol guidelines.

Isolated muscle is mounted using 10% tragacanth gum (Sigma, G1128) and quick frozen in liquid nitrogen-cooled 2-methylbutane. Samples were stored at −80°C until sectioning into 12 µm sections. Muscle cross-sections are cut using a (TBS cryostat*) set at -20°C. Sections are mounted to charged slides and allowed to air-dry at room temperature. Once dry, slides are incubated in 100% ice-cold acetone for 10 min, rinsed with water and coverslips adhered using

water-based mounting media and DAPI. Slides were then examined using fluorescence microscope (Nikon 80i upright+ and Roper Scientific EZ monochrome CCD camera with deconvolution software analysis [NIS Elements, Nikon]).

Monomeric peptide solution preparation: Synthetic crude peptides D1 wt (Desmin aa 111-143, ELQELNDRFANYIEKVRFLE QQNAALAAEVNRL), D1 AD (ELQELNDRFDNYIEKVRFLE QQNAALAAEVNRL), D2 wt (Desmin aa 269-290, DLTAALRDIRAQYETIAAKNIS), and D2 AV (DLTAALRDIRAQYETIVAKNIS) were obtained from Watsonbio. These peptides were purified through reverse-phase HPLC (Sorbax 300SDC3) on a water/acetonitrile gradient containing 0.1% trifluoroacetic acid, lyophilized and dissolved in Hexafluoroisopropanol (HFIP) at 5mg/ml for half an hour, aliquoted in tubes and lyophilized again. The aliquoted peptides were stored at 4° C. In order to prepare monomeric peptide solutions, peptides were dissolved in dry DMSO (33 mg/ml) and directly added to buffer solution (20 mM glycine buffer, pH 2.8, 100 mM NaCl).

Desmin intermediate filament formation: Full length desmin was purchased from Origene protein in lyophilized form in 4M Urea. The protein solution was reconstituted with water, dialyzed (10000 MWCO dialysis cassette, ThermoScientific) in 10 mM Tris buffer, pH 8.0, containing 50 µM TCEP overnight at 4°C. 0.1-0.3 mg/ml concentration of protein was rebuffered in 100 mM

imidazole, pH 6.8, and placed the solution at 37°C for 1 hour to produce intermediate filaments.

Fluorescent labeling and co-aggregation of desmin polypeptides: We labelled D1AD peptide with Atto 532-NHS ester (Sigma, 88793). The lyophilized peptide was dissolved in dry DMSO and 1M Na_2HCO_3 was added to the DMSO solution to a final peptide concentration of 25 mg/ml. 1.5 mol equivalents of Atto 532-NHS ester solution in DMSO (10 mg/ml) were added to peptide, incubated at room temperature for 4 h with under constant agitation with a magnetic stir bar. Synthetic IAPP peptide (Watson Bio) and desmin 117-348 polypeptide (des117) were labeled with Atto 488-NHS ester (Sigma, 41698) analogous to the D1AD peptide. Labeled IAPP peptide was purified by reverse-phase HPLC as described above.

For labeling of full length desmin protein (desmin-647) or desmin 117-348 fragment with Alexa 647 maleimide, we dialyzed the polypeptide in 20 mM Tris buffer of pH 7.8, containing 50 µM TCEP. The concentration of desmin after dialysis was 0.3 mg/mL. To a total of 200 µL desmin solution, 10 µL Alexa 647 maleimide (10 mg/mL in DMSO) was added and the mixture stirred at 4°C overnight. Excess maleimide was deactivated by adding 2 µL β-mercaptoethanol. Labeled D1AD-Atto 532 peptide (1%) was mixed with monomeric unlabeled peptide in 20 mM glycine buffer, pH 2.8, 100 mM NaCl, 37°C temperature for 70 h to prepare labeled D1AD aggregates. The aggregated D1AD peptide was ultracentrifuged for 20 min at 165000 x g and the supernatant was discarded to

remove monomeric peptide and free dye. Labeled des117-Atto 488 aggregates were prepared analogously.

Full length desmin dialysis and aggregation: 20 microgram of desmin protein (Cellsciences) was dissolved in 10 µL water. Stepwise dialysis was done against 10 mM Tris buffer, pH 8.0, containing 4 M, 2 M, and 1 M urea at room temperature for 30 min each in a 6000 MWCO dialysis tube (Sigma, PURN60030). Protein concentration was measured by diluting the protein solution in 50 mM Na-phosphate (NaP) buffer, pH 7.4. For aggregation, the protein solution in Tris buffer containing 1 M urea was diluted 10-fold in 50 mM NaP buffer, pH 7.4, 100 mM NaCl. Aggregation was done in 10 mM Tris buffer, pH 7.8, with a final urea concentration of 100 mM.

Desmin 117-348 dialysis and aggregation: Lyophilized desmin 117-348 peptide was obtained from Cloud Clone (catalog # RPA373Hu01). The polypeptide was dissolved in water and dialyzed overnight in 10 mM Tris buffer, pH 8.0 using a 6000 MWCO dialysis tube. After dialysis, concentration of the peptide calculated by measuring absorbance (ε = 14440 /M /cm). For aggregation, this peptide was directly added to 50 mM NaP buffer, pH 7.4, 100 mM NaCl or 20 mM glycine buffer of pH 2.8, 100 mM NaCl.

Recombinant desmin expression and purification

Desmin (aa residues 117-348; Accession number P17661) was prepared according to Jackson et al. 1999 with modifications. Briefly, the DNA sequences encoding Desmin 117-348 protein in pTrcHisB were transformed into BL21 (DE3). BL21 cultures were grown in LB medium in the presence of 100µg/ml Ampicillin. Expression of protein was induced using 1mM IPTG and was purified from inclusion bodies under denaturing conditions using Nickle superflow resin with an AKTA Pure (GE health care Life Sciences). The protein was eluted from the column using an imidazole gradient. The eluted material was extensively dialyzed against 25mM Tris / 2mM EDTA / 10mM DTT / 150mM NaCl pH. 8.0. Protein had Histidine tag cleaved with the addition of 2.5mM $CaCl_2$ and 50U Thrombin (VWR). Protein was run through a second NiNTA on the AKTA Pure to remove the His tag. Clean protein was dialyzed against 10mM Tris pH 8.0. The final concentration of the Desmin protein was determined by A_{280nm} with ε= 14440/M/cm as extinction coefficient. Aliquots were stored at −80°C until use.

Alpha-synuclein expression, labeling and aggregation: α-synuclein was expressed in Escherichia coli and purified as described previously (57) and then lyophilized for storage at -20°C. Alpha-synuclein was labeled with Alexa 647-NHS ester by dissolving lyophilized protein in 1M Na_2HCO_3 to a final peptide concentration of 2 mg / mL. 1.5 mol equivalents of Alexa 647-NHS ester solution in DMSO (10 mg/ml) were added to the protein, incubated at 4°C over night with under constant agitation with a magnetic stir bar. Lyophilized protein was dissolved in 10 mM NaOH to final concentration of 2 mg/mL, vortexed gently and

sonicated in a water bath at 20 °C for 15 minutes. The suspension was then centrifuged at 50,000 RPM at 4 °C for 20 minutes after which the supernatant was collected. 90 µM α-synuclein + 1 µM α-synuclein-Alexa 647 was aggregated in 100 mM Na-phosphate buffer, pH 7.4, 10 mM NaCl with a 2 mm glass bead. Aggregation kinetics were recorded on an InfinitE M200 Tecan plate reader with a shake time of 5 seconds every minute, amplitude of 1 mm in non-binding 96-well plates (Corning 3651).

Circular Dichroism: Circular Dichroism (CD) was performed in a 1mm quartz cuvette on Jasco J-810 Spectropolarimeter as described in Lam et al. 2016. The scan speed was 50 nm / minute, data pitch of 0.5, response time 8 s, 5-10 accumulations, continuous scan for collection of data for the secondary structure of peptides. The scanning range was 200 to 260 nm. Molar ellipticities were calculated by subtracting the signal of buffer control samples from the peptide spectra.

ThT aggregation assay: D1 and D2 peptides were dissolved in DMSO (10 mg / ml) and then diluted to a final concentration of 0.33 mg/ml into either 20 mM glycine buffer, pH 2.8, Sodium acetate + Sodium phosphate buffer, pH 4.0, Sodium phosphate buffer, pH 7.4, Tris-HCl, pH 8.0, Sodium borate, pH 9.0, with 100 mM NaCl. ThT dye (20 µM) was added to monitor the aggregation of the peptide. For full length desmin and des117 (CloudClone), 50 mM NaP buffer, pH

7.4, 100 mM NaCl was used. Alternatively, des117 was aggregated in 20 mM glycine buffer, 100 mM NaCl, pH 2.8. Aggregation was monitored in Tecan infinite F200 plate reader in 96-well black wall, clear bottom (Corning 3651) plates. Temperature was set to 37°C and plates were shaken for 5 seconds every 10 minutes. The excitation and emission wavelengths were 420 and 480 nm respectively. Fresh, recombinant expressed des117 was aggregated in 50 mM NaP buffer, pH 7.4, 100 mM NaCl, 20 µM ThT in a Clariostar plate reader (BMG Labtech) with 5s shaking (700 rpm, orbital) every 10 minutes at 37°C, resulting in somewhat faster aggregation kinetics than the lyophilized commercial des117 protein. ThT fluorescence was normalized to post-aggregation signals. Normalized results are reported as means ± SD (n=3); significance was $p < 0.001$ or higher comparing seeded vs. unseeded reactions (Figs. 1B, C, G; 2D, E, F; 3C, F), most significances were omitted from figures to improve legibility.

Ultracentrifugation: Ultracentrifugation assays were performed in a Beckman TL-100 ultracentrifuge, with a TLA-100 fixed angle rotor in thick walled polycarbonate 200-µL tubes (Beckman Coulter) at 70,000 rpm for 20 minutes at 4 °C. The supernatant was collected slowly from top and mixed with SDS-PAGE LDS sample buffer and boiled (95 °C) for 10 minutes before running through the SDS PAGE gel.

SDS PAGE gel: For SDS-PAGE (Biorad), samples were diluted in LDS sample buffer (Invitrogen) and heated for 10 min at 95 °C. Gel bands were visualized by

Coomassie staining (Sigma Aldrich). Gels were scanned digitally and the intensity of bands was quantified by the Image j software.

Congo Red staining: 500 μM Congo Red dye solution was prepared in water, filtered through a 0.2 μm membrane and stored at 4°C. The aggregated peptide solution was pipetted on a coverslip. Aggregates formed at low pH were rebuffered by adding 100 mM Sodium Borate buffer of pH 8.0, and Congo red solution was added to 50 μM final concentration. Samples were imaged on an inverted fluorescence microscope (Nikon Eclipse TE2000-U) using a Zeiss Axiocam 512 color camera. For fluorescence imaging, the sample was excited at 532 nm and emission was collected through a 568 nm long-pass filter. For birefringence imaging, two polarizers were angled at 90° before and after the sample, and samples were excited by white light.

Atomic Force Microscopy: Aliquots of aggregated peptides (10 μL) were placed on clean, freshly cleaved grade V-1 mica (Cat#: 01792-AB, Structure Probe, Inc., USA). After 10 minutes, the solvent was wicked off by filter paper and the mica was washed 4 times with 20 μL of water to remove salts and buffer from the sample. Samples were dried overnight, and AFM images were acquired in tapping mode on a Veeco Dimension 3100 machine (Bruker) with Bruker FESP tips. Images were visualized using the Bruker Nanoscope software v1.5, and height profiles were analyzed using the Nanoscope section tool and FFT tool.

Transmission electron microscopy: Samples (5 µL) were loaded onto carbon-coated 300 mesh copper grids (Electron microscopy Sciences) that were glow discharged for 40 seconds using an PELCO easiGLOW™ glow discharge unit (Ted Pella Inc., USA). Samples were left to bind for 1 minute, blotted dry, washed in water (3 x 30 µL), blotted, and then stained with 10 µL NanoW stain (Nanoprobes) for 1 min. Images were acquired on an FEI Tecnai T10 electron microscope (FEI, Eindhoven, NL). Dimensions of 20 fibrils per sample were measured in ImageJ using the sectioning tool. Values are reported as mean ± standard deviation.

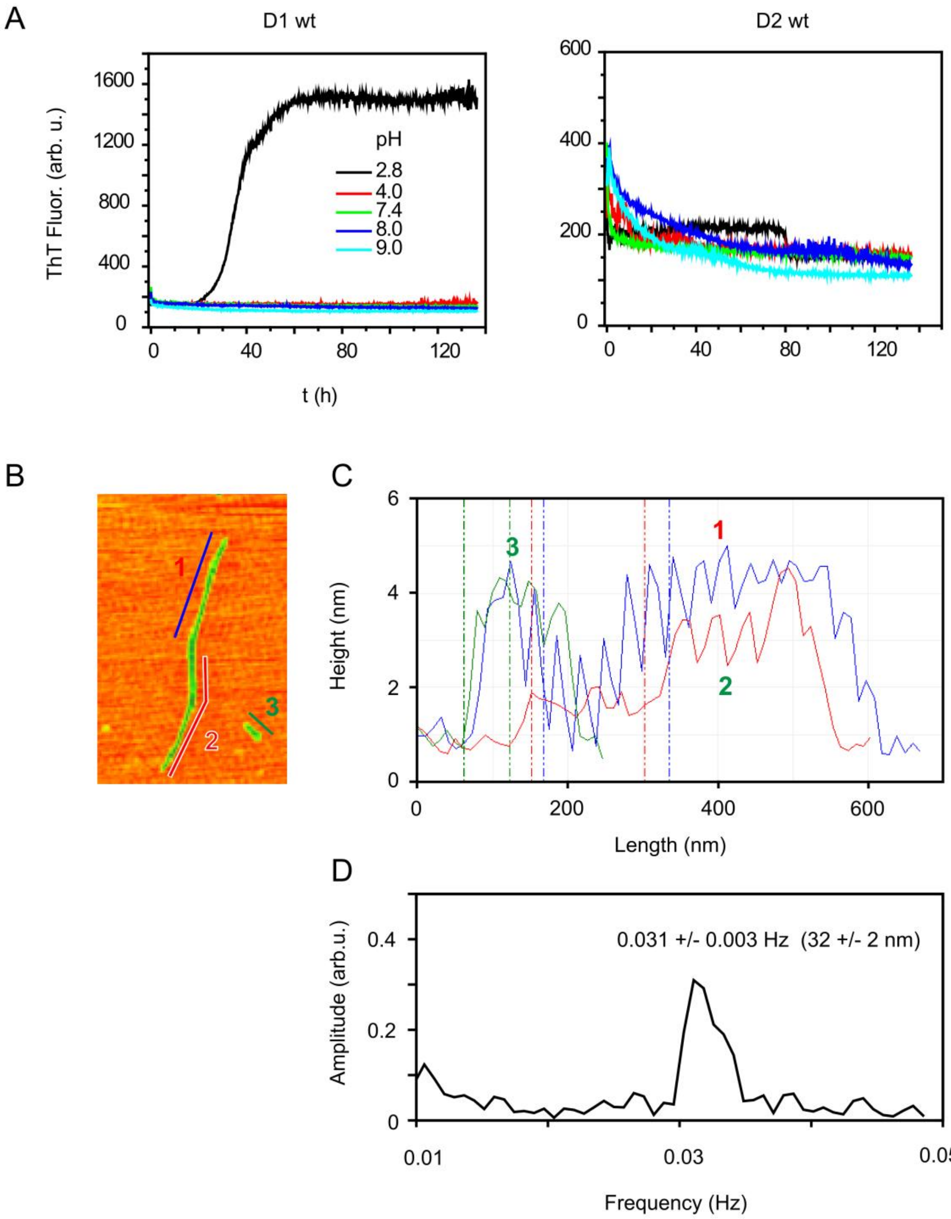

Fig. S1. A) Aggregation of D1wt and D2wt peptide in 20 mM buffer of different pH, with 100 mM NaCl. Aggregation kinetics were monitored by ThT dye at 37˚C temperature. B) High resolution atomic force microscopy image of D1wt fibril. C) Height profiles were analyzed for regions 1, 2, and 3, highlighted in blue, red, and green, respectively. D) Fourier transform analysis of height profiles 1, 2, and 3 from panel B).

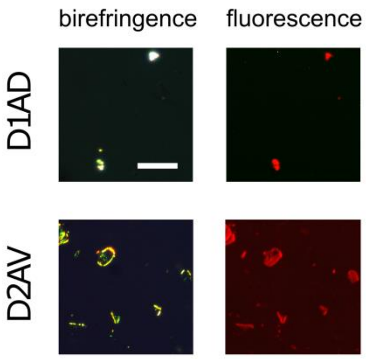

Fig. S2. Congo red fluorescence and birefringence images of D1AD and D2AV aggregated peptides in 20 mM glycine buffer of pH 2.8, 100 mM NaCl. The aggregates were rebuffered by Sodium Borate to neutral pH to visualize Congo red fluorescence; scale bar 50 μm.

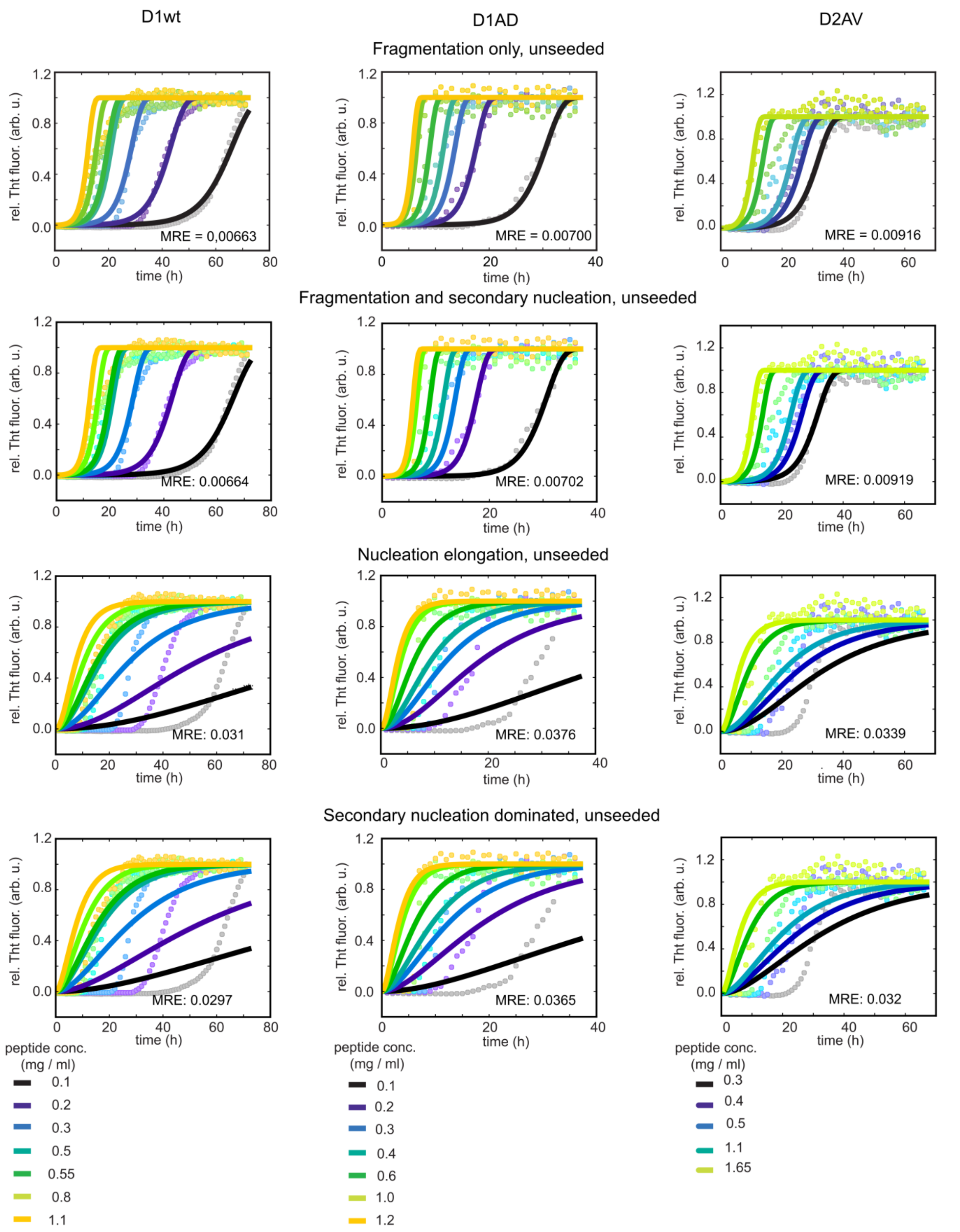

Fig. S3. Concentration dependent aggregation kinetics of D1wt, D1AD and D2AV peptides in 20 mM glycine buffer, pH 2.8, containing 100 mM NaCl were fitted using AmyloFit with three unseeded models namely fragmentation, fragmentation + secondary nucleation, nucleation elongation, Secondary nucleation dominated. All data were calculated from triplicate data sets at each peptide concentration. Plotted are averaged data (dots) and global fit results (lines).

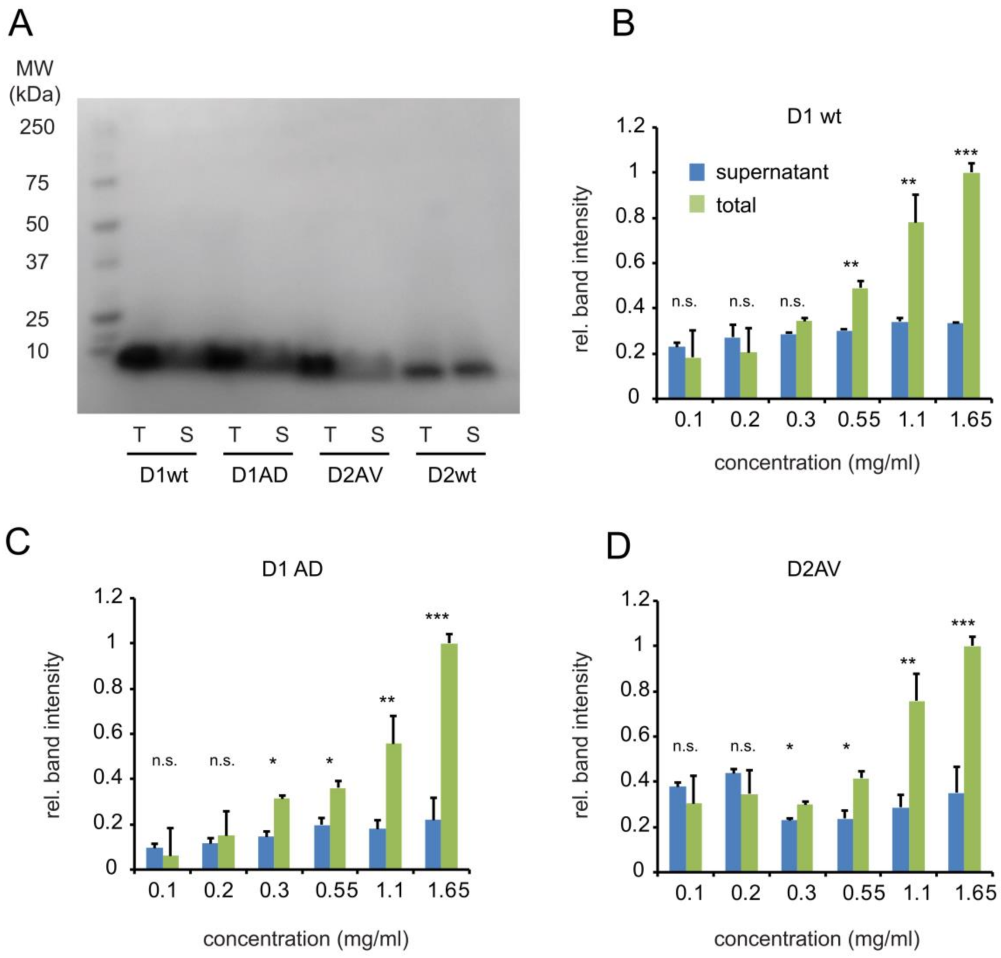

Fig. S4. Quantification of total D1wt peptide in solution and soluble peptide in the supernatant after centrifugation at 168000 x g for 20 min after aggregation in 20 mM glycine buffer, 100 mM NaCl, at pH 2.8. (A) Coomassie stained SDS-PAGE gel of total peptide (T) and soluble (S) fractions of D1wt, D1AD, D2wt and D2AV peptides after 72 h of aggregation. The initial peptide concentration was 0.33 mg/ml. (B, C, D) Soluble supernatant and total peptide concentrations calculated from Coomassie gel staining intensities; mean ± SD, n = 3. Here, peptides were aggregated for 4 days at different initial concentrations (0.1-1.65 mg/ml) and analyzed as in (A). D2 wt peptide, which remained completely soluble, was not analyzed; * indicates $p < 0.05$, ** $p < 0.001$, *** $p < 0.0001$.

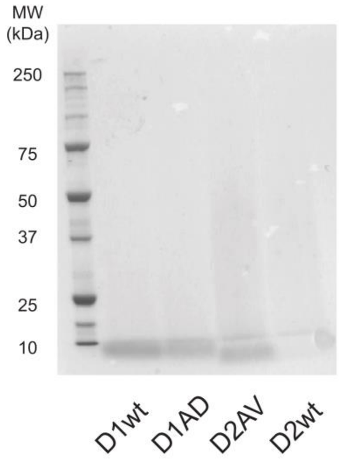

Fig. S5. SDS-PAGE analysis of desmin peptides incubated in presence of EGCG (5 x molar ratio) as described in Fig. 3 A-C. Peptides were stained by Coomassie brilliant blue. No SDS-stable high molecular weight complexes were formed either in presence of EGCG.

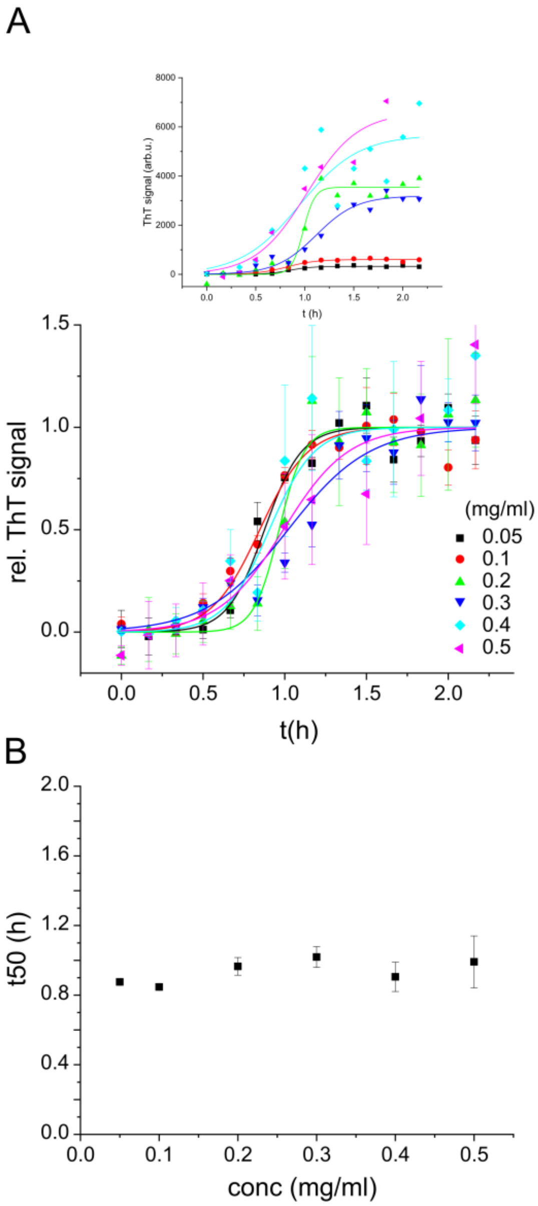

Fig. S6. (A) Aggregation of recombinant desmin 117-348 polypeptide (des117) in 50 mM nap buffer, pH 7.4, containing 100 mM NaCl. The kinetics were monitored by ThT, at 37˚C at protein concentrations between 0.05 – 0.6 mg/ml. Main panel: mean normalized ThT fluorescence ± SD, n = 3; inset: mean ThT fluorescence. Lines correspond to sigmoidal fits of fluorescence intensities by F(t) = 1/(1 + exp k(t – t50)). (B) Concentration dependence of half-maximal ThT signal (t50); mean ± SD, n = 3; t_{50} values were derived from sigmoidal fits of (A). None of the data showed significant differences in t_{50} or in normalized ThT signals.

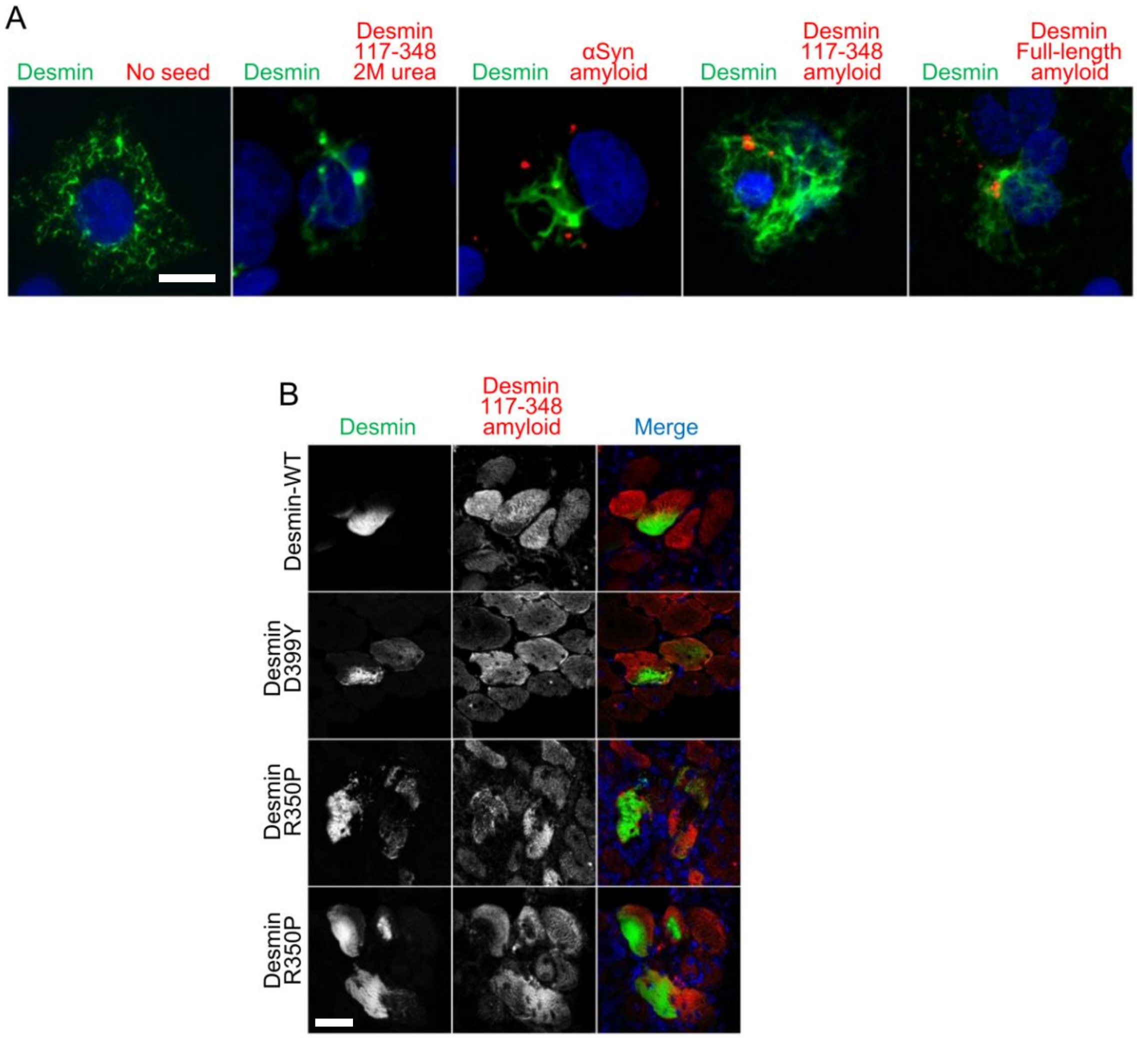

Fig. S7. (A) Confocal images of SWI3 cells transiently transfected with GFP-desmin (green) and Lipofectamine transduced with des117 in 2M urea, α-synuclein seed, des117 amyloid or full-length desmin amyloid (all fluorescently labeled with Alexa 647, shown in red); scale bar 10 µm. (B) Confocal images of cryosections of tibialis anterior muscle electroporated with GFP-Desmin-WT or one of two MFM Desmin mutants (D399Y or R350P) and fluorescently labeled with des117 amyloid; scale bar 50 µm.

References

1. Lam HT, Graber MC, Gentry KA, & Bieschke J (2016) Stabilization of alpha-Synuclein Fibril Clusters Prevents Fragmentation and Reduces Seeding Activity and Toxicity. Biochemistry 55:675-685.

2. Jackson GS, Hill AF, Joseph C, Hosszu LLP, Clarke A, Collinge J (1999) Multiple folding pathways for heterologously expressed human prion protein. Biochim. Biophys. Acta 1431: 1–13.
